# Complement C3 of the innate immune system secreted by muscle adipogenic cells promotes myogenic differentiation

**DOI:** 10.1038/s41598-017-00099-7

**Published:** 2017-03-13

**Authors:** Thierry Rouaud, Nader Siami, Tanaelle Dupas, Pascal Gervier, Marie-France Gardahaut, Gwenola Auda-Boucher, Christophe Thiriet

**Affiliations:** UFIP UMR-CNRS 6286, Epigénétique: prolifération et différenciation, Faculté des Sciences et des Techniques, 2 rue de la Houssinière, 44322 Nantes, France

## Abstract

Myogenic differentiation results in different cell type cooperation, but the molecules involved in the myogenic cell activation remain elusive. Here, we show that muscle-resident pre-adipocytes promote myogenic differentiation through the secretion of factors. Using proteomic and transcriptomic analyses, we identified that proliferative adipogenic lineage cells produce and secrete a key factor of the innate immune system, the complement C3. Cell culture experiments revealed that C3 promotes the differentiation of myogenic progenitors following internalisation of the immune molecule. These data demonstrate that the third component of the complement system, which is a pivotal factor in the immune response to pathogens, is also involved in the differentiation of myogenic progenitor cells.

## Introduction

Myogenic cell differentiation is a complex process involving the activation, proliferation and differentiation of progenitors^1, 2^. During development and regeneration, myogenic differentiation is perfectly coordinated and proceeds to form and regenerate the skeletal muscles^3, 4^. The overall cellular mechanism consisting in the proliferation and the fusion of the progenitor cells leading to multinucleated syncytia is shared throughout the life span. Upon activation, progenitor cells express specific myogenic transcription factors, such as MyoD, Myf5 and Myogenin^5^. This genetic cascade leads to the fusion of myoblasts that generates new myofibers.

It is generally believed that the myogenic progenitor cell differentiation is orchestrated by signals from the microenvironment^6^. The activation of embryonic and adult cells is promoted by factors that associate with membrane receptor, which induce signal transduction. For exemple, it has been shown that during regeneration, adult myogenic progenitor cells (satellite cells) expressed the Wnt receptor Fzd7 and Wnt7a that is up-regulated within the injured muscle possibly binds to the receptor^7^. Undoubtedly, Wnt7a plays an important role in regulating satellite cell function. However, it is likely that *in vivo* this function is redundant, as the inhibition of the Wnt/β catenin pathway only delays muscle regeneration^8^. Interestingly, Wnt signalling plays a key role in regulating developmental programs through embryonic development and in regulating stem cell function in adult tissues. In myogenic differentiation, Wnt factors have been demonstrated to be necessary for embryonic myogenic induction in the paraxial mesoderm and in the control of differentiation during muscle fiber development^9^. In adult, the Wnt signalling is required for the myogenic commitment and adult stem cells in muscle tissue following injury^8^.

Although myogenic factor, such as Wnt7a is secreted in injured muscle, the actual cellular origin remains elusive. Furthermore, beside the Wnt pathway, it has been shown that other cytokines affected myogenic differentiation^10^. Recently, it has been shown that undifferentiated cells of the adipogenic lineage promote myogenic differentiation of progenitor cells in a cell-to-cell contact-independent manner^11^. In contrast, following adipocyte differentiation, the formation of myotube was limited. However, the factors involved in myogenic differentiation are undetermined. Similarly, we have shown that CD34^+^ cells isolated from fetal mouse muscles, which can regenerate adult injured muscle, display distinct sub-populations presenting different differentiation characteristics (e.g. adipogenic, angiogenic and myogenic lineage)^12^. Nonetheless, myogenic regeneration was enhanced when myogenic lineage cells were transplanted with adipogenic and angiogenic cells. These studies strongly suggested that myogenic differentiation results in cooperation between different cell lineages, but did not allow the identification of the factors that promote the differentiation^11, 12^.

In the present study, we report a novel myogenic factor secreted by undifferentiated preadipocyte that enhances myogenic differentiation of fetal progenitor cells and adult cells. Unexpectedly, the myogenic factor is related to innate immune system, namely complement C3. We showed that complement C3 molecule internalizes myogenic and adipogenic precursor cells and then promotes their differentiation. However, our analyses suggested the presence of C3 is favourable to myogenesis rather than adipogenic differentiation, since myogenic progenitor cells differentiate faster than preadipocytes to adipocytes.

## Results and Discussion

### Preadipocyte, but not adipocyte promotes myogenic differentiation via secreted factors

**Figure 1:**
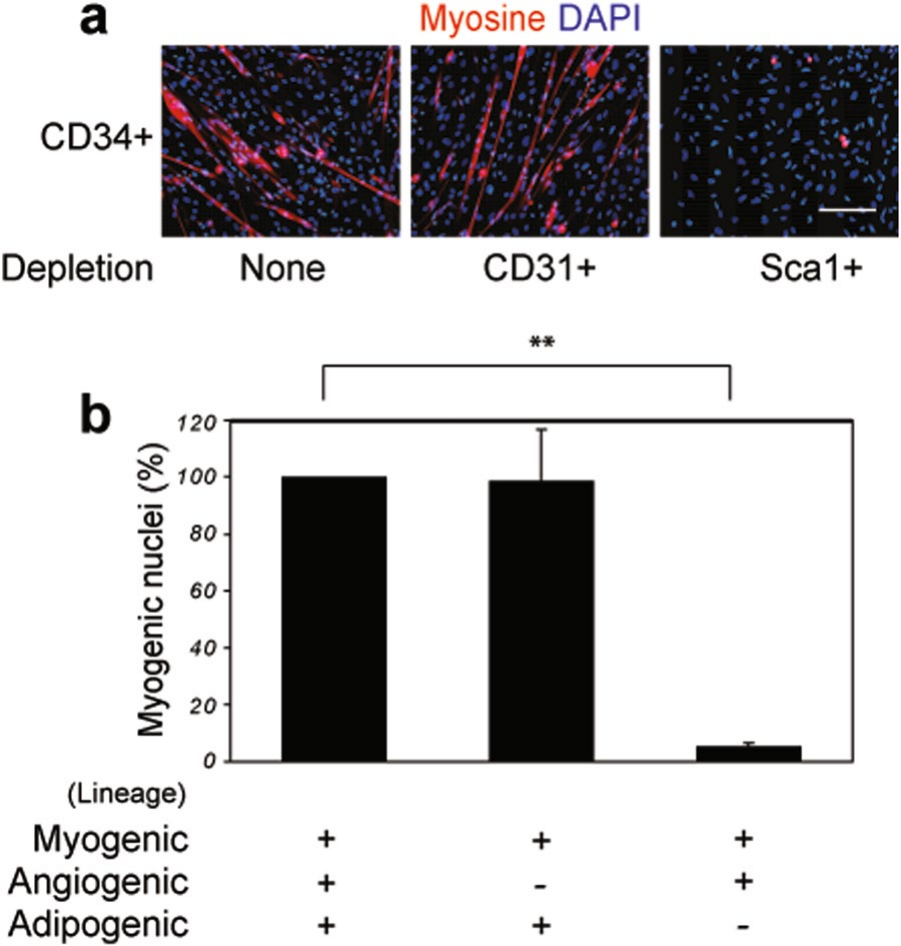
Adipogenic lineage cells are required for myogenic differentiation of competent cells. (**a**) Microscopic observations of the myogenic differentiation of competent cells. Cells harvested from leg muscles of 17 dpc foetuses and sorted into a CD34^+^ population (CD34^+^/depletion none), CD34^+^/CD31^−^ cells (CD34^+^/depletion CD31^+^), and CD34^+^/Sca1^−^ cells (CD34^+^/depletion Sca1^+^), respectively. The different cell groups were cultured for 4 days and myogenic differentiation was assessed by immuno-staining using an anti-myosin antibody (My32, red), counterstained with DAPI (blue). Bar scale corresponds to 100 μm. (**b**) Quantitative analyses of myogenic differentiation of competent cells. The percentage of myogenic differentiation was determined microscopically. Total CD34^+^ cell population was used as a reference and myogenic differentiation was arbitrarily estimated at 100%. Statistics: n = 3, t-test P < 0.0001 (**).

Studies have suggested that myogenic differentiation results in cooperation between different cell lineages^12, 13^. CD34^+^ mouse foetal muscle cells exhibit muscle regeneration properties and are composed of three distinguishable lineages; myogenic, angiogenic (CD34^+^/CD31^+^) and adipogenic (CD34^+^/Sca1^+^)^12, 14^. Using cell cultures, we determined whether myogenic differentiation of CD34^+^ foetal muscle cells is affected by the absence of the angiogenic sub-population (CD34^+^/CD31^+^) and the adipogenic sub-population (CD34^+^/Sca1^+^) (Fig. 1). We found that cultures with similar growths exhibited a significant decrease of the myogenic differentiation in absence of cells of the adipogenic lineage. Our results with foetal cells are consistent with those of adult cells, showing that adipogenic cells affect myogenic differentiation in a cell-to-cell contact-independent manner^11, 13^.

**Figure 2:**
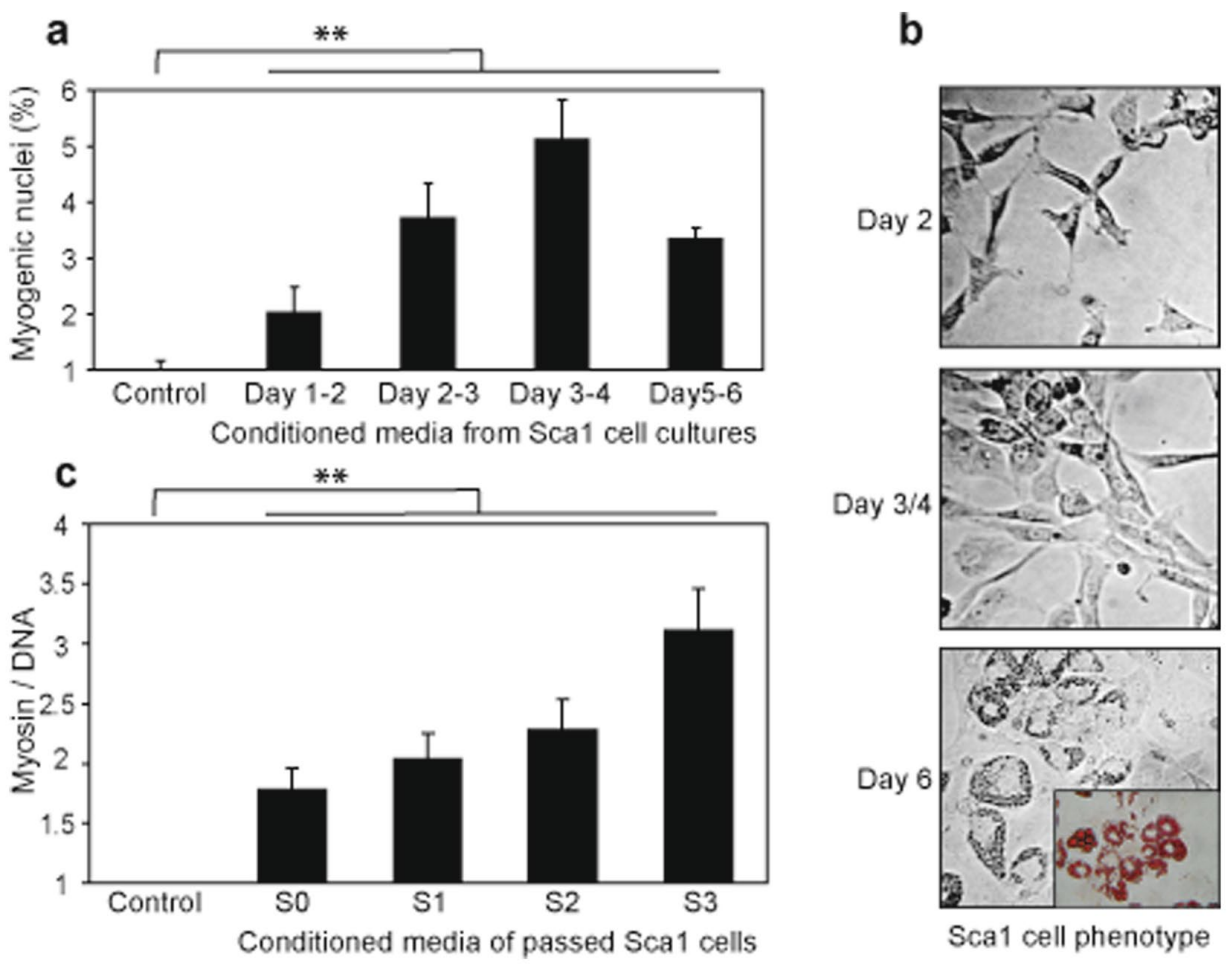
Proliferative pre-adipocytes produce myogenic factors. (**a**) Conditioned media prepared from CD34^+^/Sca1^+^ primary cell cultures enhanced myogenic differentiation of initial fraction cells. Conditioned media were prepared from 24 h of CD34^+^/Sca1^+^ cell cultures between day 1–2 (Day 1–2), day 2–3 (Day 2–3), day 3–4 (Day 3–4) and day 5–6 (Day 5–6), respectively. The percentage of myogenic nuclei was assessed microscopically on initial fraction cells cultured in conditioned media for 4 days, using cells in DMEM/decomplemented FCS as a control (Control). (**b**) CD34^+^/Sca1^+^ cell phenotype in cell culture. Isolated CD34^+^/Sca1^+^ cells (Sca1) were cultured in DMEM/decomplemented FCS and micrographs were taken after 2 days (Day 2), 3–4 days (Day 3/4) and 6 days (Day 6), respectively. Insert in (Day 6) represents Oil Red O staining of differentiated adipocytes. (**c**) Conditioned media from proliferative CD34^+^/Sca1^+^ cells enhanced myogenic differentiation. CD34^+^/Sca1^+^ primary cells were cultured and passaged just prior to confluence. Conditioned media were prepared between day 3 and 4 of primary cell culture (S0), after 1 passage (S1), after 2 passages (S2), and after 3 passages (S3), respectively. The myogenic differentiation was estimated by fluorescence (see details in materials and methods). Statistics: n = 3, t-test P < 0.0001 (**).

To determine whether molecules from adipogenic lineage stimulate myogenic differentiation of precursors, conditioned media from adipogenic lineage cell cultures were prepared and assayed for effect on cells harvested from the foetal initial fraction (the initial fraction corresponds to individual cells harvested from muscle) (Fig. 2a,b). Analyses of myogenic differentiation were performed by counting the percentage of myogenic nuclei. The results revealed an increase of myogenic nuclei when the initial fraction was cultured in the conditioned media prepared from a proliferative adipogenic lineage culture (Day 1–2 to Day 3–4). Beyond Day 4–5, the CD34^+^/ Sca1^+^ cell cultures reached confluence and began to differentiation into adipocyte and the resulting conditioned exhibited a decrease of myogenic nuclei (compare Day 3–4 and Day 5–6). These results suggested that myogenic effect of the conditioned media correlates with the proliferative and undifferentiated states of the CD34^+^/Sca1^+^ cells. To verify that the proliferation of the undifferentiated CD34^+^/Sca1^+^ cells supported the myogenic effect, we prepared conditioned media of cultured CD34^+^/Sca1^+^ and performed passages of the cells after 4 day-cultures, prior to reach confluence for allowing cell expansion (Fig. 2c). The analyses of the conditioned media prepared from the different passages showed that myogenic effects are recovered over three passages of the CD34^+^/ Sca1^+^ cells, above this number of passages, CD34^+^/Sca1^+^ cells did not proliferate and myogenic effect was significantly reduced (data not shown). Unexpectedly, we reproducibly noticed that the passages of the CD34^+^/Sca1^+^ cells improved the myogenic effects of the conditioned media. Supposedly, the passage of the CD34^+^/Sca1^+^ cells induced selection. However, because the myogenic effect might be due to different proliferation rates of the myogenic lineage, we examined the kinetics of proliferation in presence of control media and conditioned media (Supplementary Fig. 2). The results revealed that the cell proliferation is unaffected by the conditioned media as similar rates of proliferation were observed in control and conditioned media. We concluded that conditioned media from proliferative adipogenic lineage sustain myogenic differentiation effects. These data support the idea that muscle-resident undifferentiated adipocytes synthesize molecules that promote myogenic differentiation^13^.

### Analyses of the preadipocyte secretome

**Figure 3:**
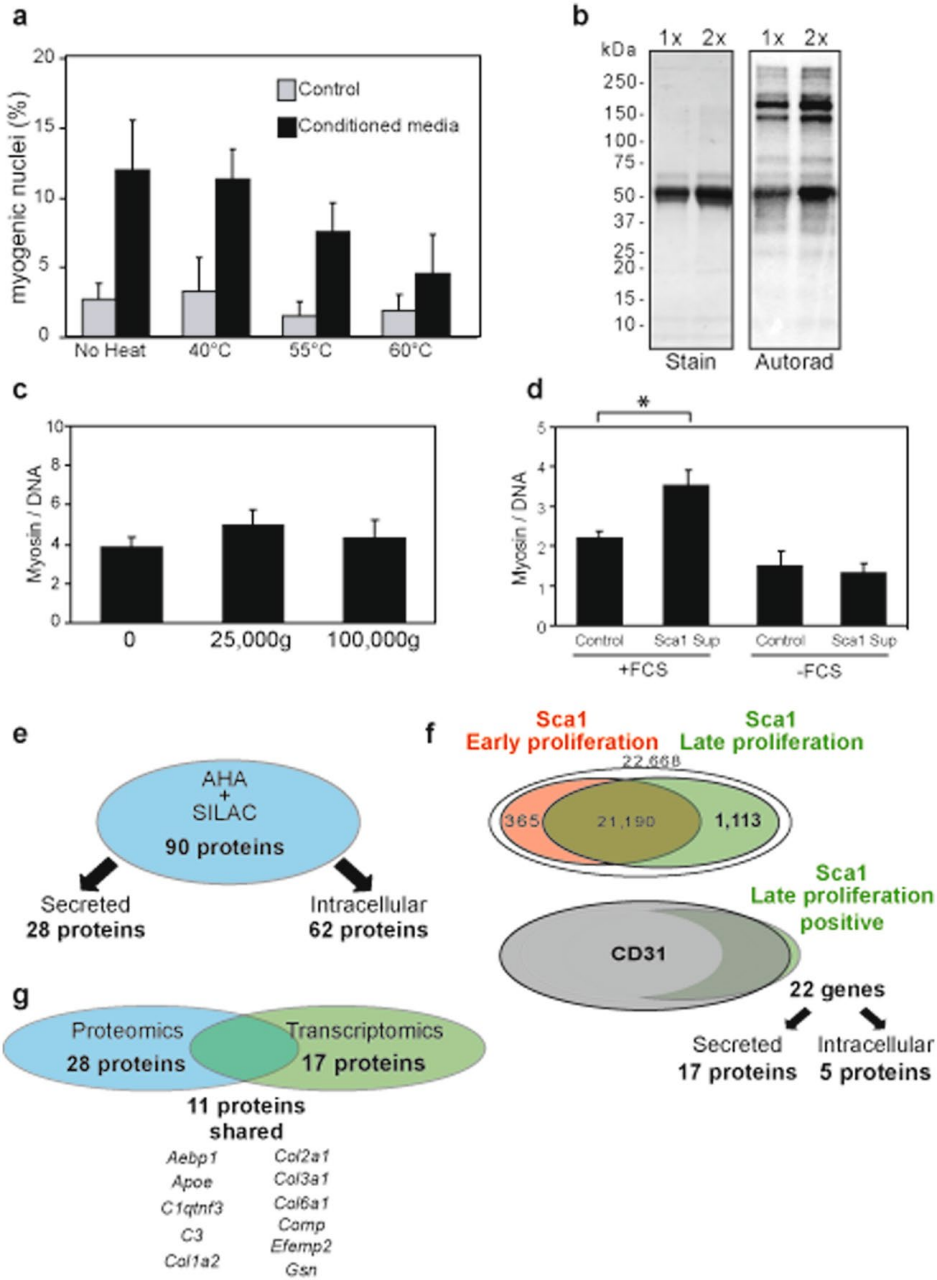
Identification of the putative myogenic factors produced by undifferentiated adipocytes. (**a**) Myogenic factors of the conditioned media are thermo-sensitive. Control and conditioned media were incubated at different temperatures for 15 min. The myogenic potential of the media was evaluated microscopically on the in initial fraction. (**b**) CD34^+^/Sca1^+^ cells secrete proteins. CD34^+^/Sca1^+^ cells were pulsed with tritiated lysine for 24 h and the culture media were analyzed by SDS-PAGE (Stain) and autoradiography (Autorad). (**c**) Myogenic factors secreted by CD34^+^/Sca1^+^ cells are soluble. Conditioned media were centrifuged at 25,000 g and 100,000 g, respectively. The myogenic potential of the resulting media was then evaluated using non-centrifuged conditioned medium as a control (0). (**d**) CD34^+^/Sca1^+^ cell cultures require FCS to produce efficient conditioned media. Conditioned media were prepared from CD34^+^/Sca1^+^ cultured in DMEM/decomplemented FCS (+FCS) and DMEM (−FCS), respectively. The myogenic potential of the resulting conditioned media was then evaluated using as control DMEM/decomplemented FCS (+FCS) and DMEM (−FCS), respectively. Statistics: n = 3, t-test P < 0.001 (*). (**e**) Proteomic analyses of CD34^+^/Sca1^+^ primary cell conditioned media. Proteins were prepared as described in Methods, identified by mass spectrometry and sorted by their cellular location; extracellular (Secreted) and intracellular (Intracellular). (**f**) Transcriptomic analyses of CD34^+^/Sca1^+^ primary cells. Transcriptomes of CD34^+^/Sca1^+^ cells were analyzed in early proliferative state (lower efficiency of the secretome, lower rate of proliferation, Sca1 Early proliferation) and late proliferative state (higher efficiency of the secretome, higher rate of proliferation, Sca1 Late proliferation), respectively. The positive probes were sorted by the level of expression in the two culture states, arbitrary considering 2-fold changes as over-expression. The probes with higher expression in proliferative state were then analyzed against the transcriptome of CD34^+^/CD31^+^. The over-expression in CD34^+^/Sca1^+^ cells (Sca1 Late proliferation positive) relative to CD43+/CD31^+^ cells (CD31) was more than a 2-fold change. The gene products were then sorted by their cellular location; extracellular (Secreted) and intracellular (Intracellular). (**g**) Cross-analyses of proteomics and transcriptomics revealed putative myogenic candidates. Comparison of secreted proteins from proteomic and transcriptomic analyses revealed 11 proteins in common.

To investigate the myogenic factors from pre-adipocytes, we first attempted to determine their biochemical nature. Conditioned media were thus subjected to RNase treatment and heat prior to evaluating the residual myogenic activity. The conditioned media were unaffected by RNase treatment (Supplementary Fig. 3), but were sensitive to heat treatment, suggesting that the myogenic factors are proteins (Fig. 3a). To confirm the presence of proteins from proliferative adipogenic lineage within the conditioned media, the cells were pulse-labelled with tritiated lysine and the culture media were examined by autoradiography (Fig. 3b). Only a limited number of proteins were radioactively labelled, suggesting that the myogenic factors from adipogenic lineage are secreted and did not result from cell lysis. These proteins were synthesized in low abundance as no specific bands could be detected by gel staining (Supplementary Fig. 4). Next, we investigated whether the myogenic factors from the adipogenic lineage recovered in the conditioned media were soluble or packaged in micro-vesicles, as described for the myoblast secretome^15^. Conditioned media were spun at high speed to pellet micro-vesicles and the supernatant was assayed for its capacity to promote myogenic differentiation (Fig. 3c). Fluorescence of myosin/DNA of initial fraction cultured with the soluble fractions were similar to that with the conditioned media. These results showed that proteins of the conditioned media involved in myogenic differentiation are secreted from the proliferative adipogenic lineage.

To identify the myogenic factors of the conditioned media, proteomic analyses of the secretome of the adipogenic lineage were carried out. Typically for such analyses, cells are grown in the absence of serum^15, 16^. We therefore verified that the cells of the adipogenic lineage proliferated and secreted the myogenic factors in the absence of serum. CD34^+^/Sca1^+^ cells were cultured in the presence and in the absence of decomplemented FCS, and the culture media were harvested to prepare the conditioned media as earlier. Then, the conditioned media were assayed for their ability to enhance myogenic differentiation of initial fraction cells (Fig. 3d). Consistent with our previous analyses, we found that myogenic differentiation with conditioned media from CD34^+^/Sca1^+^ cells in presence of decomplemented FCS is about 2 fold greater than control cultures (see Fig. 2c, control vs S0). However, when decomplemented FCS was omitted CD34^+^/Sca1^+^ cell cultures, the resulting conditioned media failed to enhancement the myogenic differentiation. These results showed that CD34^+^/Sca1^+^ cells secreted myogenic factors when cultured in presence of decomplemented FCS, and thus proteomic analyses CD34^+^/Sca1^+^ cell secretome required FCS in the cell cultures. To overcome this limitation, the cells of adipogenic lineage were pulse-labelled with azidohomoalanine (AHA), a non-natural amino acid that can substitute for methionine, and concomitantly, non-radioactive heavy lysine was added to the culture media^17^. The secretome proteins were thus purified by coupling the AHA-containing proteins with DBCO magnetic beads. The covalently attached proteins were then analyzed by LC-MS/MS and the heavy lysine-containing proteins were selected (Fig. 3e and Supplementary Fig. 5). Of the 90 proteins that were identified, only 28 were readily secreted from cells (Supplementary Table 1).

To ensure that the identified proteins of the adipogenic lineage cell secretome were readily associated with the proliferative state of the cells, whereby the effect on myogenic differentiation has been observed, transcriptomic analyses were carried out (Fig. 3f). Adipogenic lineage cells in early and late proliferation were thus compared. 1,113 probes, corresponding to 749 genes, were up-regulated in late proliferation (Supplementary Table 2). To narrow down rationally the number of genes that might be associated with myogenic differentiation enhancement, the expression of these 749 genes in late proliferation of adipogenic lineage cells was compared with that in angiogenic lineage cells, which have no effect on myogenic differentiation (see Fig. 1). Of the 749 genes, only 22 were up-regulated in the adipogenic lineage cells. Again, we verified that gene products were secreted from cells and found that 17 gene products were present in the extracellular medium (Supplementary Table 3).

We reasoned that the factors involved in the enhancement of myogenic differentiation are probably highlighted in both proteomic and transcriptomic analyses. The proteins shared between the 28 proteins resulting from the proteomic analyses and the 17 gene products resulting from the transcriptomic analyses were thus determined. Eleven proteins met these criteria and could be putative candidates for myogenic differentiation (Fig. 3g).

### Complement C3 promotes myogenic differentiation

**Figure 4:**
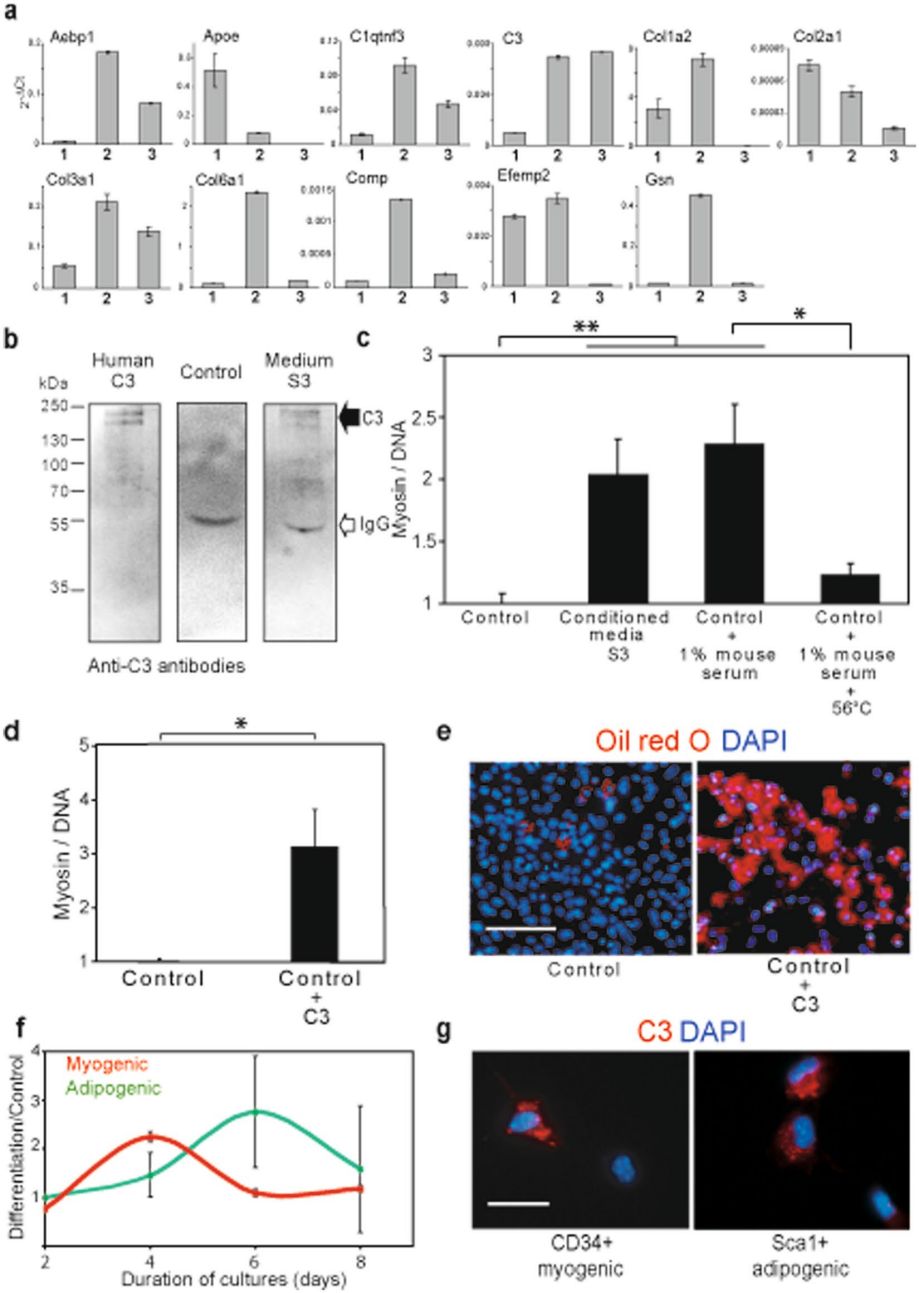
Complement C3 promotes cell differentiation of myogenic lineage cells and adipogenic lineage cells. (**a**) Expression of the putative myogenic factors in distinct cell cultures. The expression of the putative myogenic candidates identified from the omics analyses was analysed by q-RT-PCR. Three distinct cultures were examined, initial fraction cells (1), CD34^+^/Sca1^+^ primary cells (used in omics analyses and efficient for myogenic differentiation) (2), and CD34^+^/Sca1^+^ cells after three passages (highly efficient for myogenic differentiation, S3 in Fig. 2c) (3). The data are expressed relative to *Gapdh* transcription (2^−ΔCt^). (**b**) Complement C3 is present in conditioned media. Purified human C3, control media (DMEM/decomplemented FCS) and conditioned media S3 (see Fig. 2c) were resolved in SDS-PAGE and analyzed by western blotting with anti-C3 antibodies. (**c**) Growth media supplemented with serum (active complement) promoted myogenic differentiation. Initial fraction cells were cultured in DMEM/decomplemented FCS (Control), myogenic conditioned media S3 (Conditioned media S3), DMEM/decomplemented FCS supplemented with 1% mouse serum (Control + 1% mouse serum) and DMEM/decomplemented FCS supplemented with 1% mouse serum heated at 56 °C (Control + 1% mouse serum + 56 °C). After 3 days, myogenic differentiation was quantified and the control was indexed to 1. (**d**) Complement C3 promoted myogenic differentiation. Primary cells were cultured in DMEM/decomplemented FCS (Control), and DMEM/decomplemented FCS supplemented with human C3 (Control + C3). Myogenic differentiation of the different cultures was assessed relative to the control indexed to 1. (**e**) Culture media supplemented with C3 promote adipogenic differentiation. CD34^+^/Sca1^+^ cells were cultured in DMEM/decomplemented FCS (Control) and C3-supplemented control media (C3), respectively, for 6 days. Adipogenic differentiation was determined by Oil Red O staining, counterstained with DAPI. Bar scale corresponds to 100 μm. (**f**) Myogenic and adipogenic differentiation reveal different kinetics. The differentiation of adipogenic and myogenic lineage cells was estimated relative cells cultured in control media (**g**) C3 is internalized in myogenic and adipogenic cells. Fluorescently-labelled complement C3 was added to cultures of myogenic lineage cells (CD34 myogenic), and adipogenic lineage cells (Sca1 adipogenic), respectively, and observed microscopically after 4 h. Bar scale correspond to 20 μm. Statistics: n = 3, t-test P < 0.001 (*), P < 0.0001 (**).

We have shown that the secretome from adipogenic lineage cells preserves the myogenic effect as long as the secretory cells are proliferative and do not engage in adipogenic differentiation (see Fig. 2). We thus reasoned that the factors involved in myogenic differentiation are synthesized in primary cells of adipogenic lineage and cells resulting from serial passages. Therefore, the expression of the genes encoding for the putative candidates involved in myogenic differentiation was examined (Fig. 4a and Supplementary Table 4). Strikingly, the gene encoding for the third component of the complement system (C3), a key part of the innate immune system^18^, was expressed in the primary adipogenic lineage cells as well as in the proliferative cells following several passages. In contrast, all the other genes exhibited a drastically lower expression after the passages than the primary adipogenic lineage cells. We then verified by western blotting that C3 was secreted into the conditioned media (Fig. 4b). Immunodetection using an antibody to human C3 revealed the presence of bands that are consistent with C3 and in the same molecular weight range as the band detected by pulse-labelled tritiated lysine (see Fig. 3b).

To confirm that C3 is involved in enhancing myogenic differentiation, we examined whether supplementing culture media with C3 led to such an enhancement. As molecules of the complement are known to be abundant in serum, culture media were supplemented with 1% mouse serum and the effect on myogenic differentiation was examined (Fig. 4c). The analyses of the cell cultures revealed that the addition of mouse serum in the culture stimulated myogenic differentiation. Further evidence for a role of components of the complement in myogenic differentiation was revealed by the loss of the myogenic effect when mouse serum was heated to 56 °C to inactivate the complement. To ensure that C3 is responsible for myogenic differentiation, the effect on myogenic differentiation of precursor cultures supplemented with purified C3 was examined (Fig. 4d). As C3 protein presents high homology between mouse and human, these experiments were carried out with human C3 (77% identity, 92.7% similar, in 1666 aa). Similarly, the efficiency of differentiation in an adult cell culture (C2C12 cell line) was greater when human C3 was present in culture media (Supplementary Fig. 6). These results demonstrate that myogenic differentiation is enhanced by C3 protein.

Analyses of the effects of C3-containing media were also carried out directly on adipogenic lineage cells (Fig. 4e). CD34^+^/Sca1^+^ cells differentiated faster than control cultures with media supplemented with C3. Thus, C3 enhanced adipogenic differentiation. Comparison of the kinetics of differentiation of myogenic lineage cells and adipogenic lineage cells induced by C3 and relative to the respective controls revealed significantly faster myogenic differentiation than adipogenic differentiation (Fig. 4f). These observations led us to hypothesize that in the muscle environment, wherein myogenic lineage and adipogenic lineage among other lineages, C3-induced differentiation promotes myogenesis faster than adipogenesis. Moreover, these results suggested a putative autocrine signalling mechanism, as C3 production by pre-adipocytes is down-regulated by the induction of adipogenic differentiation by C3 itself.

It is generally believed that cytokines induce cell differentiation through the activation of signalling pathways. We thus examined whether such pathway activation accounted for the effect of C3 on myogenic differentiation. Common signalling pathways were scanned by antibody arrays, which showed no activation of a specific signalling pathway by C3 (Supplementary Fig. 7). It has been reported that C3 can activate immune cells by internalization^19^. Fluorescently-labelled C3 was added to culture media and its cellular location was determined by microscopy (Fig. 4g and Supplementary Fig. 8). We clearly observed that C3 molecules were rapidly internalized into the cytoplasm of myogenic CD34^+^ cells and adipogenic CD34^+^/Sca1^+^ cells. These results show that differentiation induced by C3 involves its internalization into the cytoplasm.

## Conclusion

Although different cytokines have been characterized for their involvement in myogenic differentiation, our work demonstrates that the complement C3 produced by muscle-resident pre-adipocytes promotes myogenic differentiation. Our analyses provide insight into the molecular mechanism involving C3 in myogenesis. Indeed, our results of supplementation of fluorescently labelled C3 revealed a rapid internalization of C3 within the cytoplasm prior to cell differentiation. The data suggest that muscle formation, regeneration and repair require muscle-resident cells to communicate with one another in a coordinated manner. If one cell type of this partnership is defective, muscle healing is impeded and degeneration initiated. Although the complement C3 has been detected in the vicinity of healing muscle, its role has been associated with an immune function^20^. Our findings confirm the concept that a molecule of the innate immune system facilitates non-immune cell differentiation, as it was reported for C1q, which can activate canonical wnt signalling pathway in contrast to C3.

## Methods

### Animals and isolation of foetal cells

All experiments were carried out in accordance with guide-lines approved by the University of Nantes, Institutional Animal Care and Use Committee. Adult swiss mice were maintained in our laboratory under controlled environmental conditions and fed with food and water *ad libidum*. The pregnant mice were sacrificed by cervical dislocation. Muscle cells were obtained from all four legs of 17-dpc mouse foetuses as previously described^12^. One experiment is carried out from about 30 foetuses from two or three pregnant mice. Briefly muscles were removed from the legs, minced into small fragments and digested in collagenase I (Gibco) 0.1% in Dulbecco’s modified Eagle’s medium (DMEM) (Gibco) with 1% penicillin/streptomycin/fungizon (Gibco) and 0,05% fungizon (Gibco) for 1.5 h at 37 °C. Upon sequential filtering through 70 µm and 40 µm cell strainers (BD-Falcon), the cells were collected by centrifugation at 350 g and resuspended in DMEM/decomplemented fetal calf serum (10%) (DMEM/decomplemented FCS). Resulting cells corresponding to the initial fraction of the muscle cells were checked for viability using trypan blue exclusion dye and counted in a hematocytometer.

### Immunomagnetic-bead cell sorting

Cells were sorted by MACS according to the manufacturer’s instructions (Miltenyi Biotech). For CD34^+^ sorting the freshly isolated cell suspensions were incubated for 12 min at 4 °C with a biotinylated rat anti mouse CD34^+^ antibody (clone RAM34, eBioscience 13-0341-85) 1/50 in PBS/EDTA (2 mM)/fetal calf serum (10%) (PEF) rinsed in PEF then magnetically labelled for 17 min at 4 °C with anti-biotin-coated MicroBeads (1/25 in PEF) (Miltenyi Biotech 130-090-616). Then cells were rinsed twice in PEF and MACS was performed by passing the cells over cell-separation columns (Miltenyi Biotech 130-042-201). In order to obtain CD34^+^ cells subset depleted either CD31^+^ or Sca1^+^ cells, in a first step the freshly isolated cell suspensions were incubated with PE-conjugated rat anti-mouse CD31 (clone 390, Southern Biotech 1625-09) or PE-conjugated rat anti-mouse Sca1^+^ antibodies (clone D7, eBioscience 12-5981-83) diluted 1/50 and the CD31^+^ and Sca1^+^ cells were collected using anti-PE MicroBeads (Miltenyi Biotech 130-048-801). In a second step the negative fractions for CD31^+^ or Sca1^+^ were incubated with the biotinylated rat anti mouse CD34^+^ antibody and processed as described above to obtain CD34^+^/CD31^−^ or CD34^+^/Sca1^−^ cells populations. The cells of each subset were collected by centrifugation at 350 g and resuspended in DMEM/decomplemented FCS and counted. The quality of the sorting is routinely examined by immunofluorescent microscopy (Supplementary Fig. 1).

### Cell culture in standard conditions

Cell cultures were carried out in DMEM/decomplemented FCS and used as a control, otherwise indicated. The different cell subsets (foetal cells, CD34^+^, CD34^+^/CD31^−^, CD34^+^/Sca1^−^ cells and foetal Sca1^+^ cells) were plated at 20,000 cells per cm^2^ either on 35 mm tissue culture treated dishes (Falcon 353001), on collagen-coated 8-chamber tissue culture glass slides (BD Falcon 3541108) or on collagen-coated 96 well cell culture plates (Greiner bio-one 655180). Cultures were maintained in a humidified atmosphere at 5% CO_2_ at 37 °C.

### Preparation of conditioned media

Foetal CD34^+^/Sca1^+^ cells were first grown in DMEM/decomplemented FCS on 35 mm dishes plated at 28,000 cells per cm^2^ from 2 days to 6 days and the culture medium was changed every day. In a first series of experiments the Sca1 conditioned media (S0) were prepared from the supernatant of 24 h of cell culture between day 1–2, day 2–3, day 3–4 and day 5–6. The fourth day of culture Sca1 cells were near confluence, at this stage a second series of experiments were done, cells were harvested using a trypsin digestion (trypsin-EDTA) then were grown on new dishes in DFCS in the same conditions as above. Conditioned media from this first passage (S1) were prepared from the supernatant of 24 h of cell culture between day 3–4. Then two successive passages were performed in a similar manner to collect S2 and S3 conditioned media. All the supernatants were filtered upon 0,22 μm and frozen at −80 °C.

### Cells culture in conditioned media

Cells were directly plated at the same conditions as described for the commercial medium with the conditioned media S0 to S3.

### Determination of myogenic differentiation

Myogenic differentiation of the cells was determinate after 4 days of culture by immunochemical analyses. The cultures in 96-well plates or in 8-well slides were fixed for 10 min with 4% paraformaldehyde in PBS (PAF), rinsed in PBS, permeabilized with 5% Triton X-100 (Sigma) 1 h at 37 °C, rinsed twice with PBS and then incubated overnight at 4 °C with MY32, an Ab for myosin heavy chain (1/1000, Sigma), then revealed 1 h at room temperature by FITC-conjugated goat anti-mouse Ab (1/100, Clinisciences). Nuclei were counterstained with bis-benzimide. Quantitative analysis of myogenic differentiation were carried out by quantifying myosin with MY32 antibody and nuclei with DAPI staining in 96-well plates cultures using a fluorometer (POLARstar Omega - BMG labtech). Myogenic differentiation was expressed by the ratio of the FITC myosin signal/Bis-Benzimide DNA signal. Quantification was based on two to four independent experiments with at least four to six wells each. The percentages of myogenic nuclei were quantifying directly from microscopic observations (Nikon Ni-E microscope) by calculating the ratio of the nuclei within myosin expressing cells/the total number of nuclei/cm^2^ using ImageJ software (http://rsb.info.nih.gov/ij/). Two to four independent experiments with at least three to four wells were done and four microscopic fields per well were counted.

### Determination of adipogenic differentiation

Cell cultures were rinsed 5 min in PBS, fixed with (PFA) for 20 min, washed three times with distilled water and stained in oil Red O solution for 30 min at 37 °C and then washed five times in distilled water^21^. Nuclei were counterstained with bis-benzimide. Quantification of adipogenic differentiation was done from microscopic determinations of the lipid accumulations by quantifying the areas stained by the red oil per cm^2^. The results were expressed as the ratio of lipid surface/number nuclei per cm^2^. Two independent experiments with two wells were done and ten microscopic fields per well were counted.

### RNA analyses

Total RNA was isolated using Trizol (Invitrogen) and the absence of DNA was determined by PCR. Reverse transcription reactions were performed using the iScript cDNA synthesis kit according to the manufacturer’s instructions (Bio-Rad). RT-PCRs were performed using appropriate primer sets (supplementary Table 1) and the Maxima SYBR green qPCR master mix according to the manufacturer’s instructions (Fermentas), and analyzed using Bio-Rad CFX manager (Bio-Rad) using *Gapdh* as a reference. Transcriptomic analyses were performed using an Agilent mouse expression array according to the manufacturer’s instructions.

### Protein analyses

Electrophoretic analyses were carried out according to standard protocols. Pulse-labelling with tritiated lysine was performed for 24 h by adding 0.5% (v/v) of L-[4,5-^3^H(N)]-Lysine (Specific Activity: 80–110 Ci/mmol) to DMEM/decomplemented FCS. Complement C3 was immunodetected using anti-C3 antibodies (Origen). Samples were prepared for mass spectrometry analyses as previously^17^ with minor modifications. The culture medium was supplemented with 0.8 mM heavy Lysine and 0.4 mM AHA. Click chemistry was carried out using dibenzocyclooctyne (DBCO) magnetic beads according to the manufacturer’s instructions (Jena Bioscience). The beads were then extensively washed with urea and SDS and the coupling of proteins to beads was examined by addition of fluorescein-5-maleimide and SDS-PAGE. Complement C3 was fluorescently labelled by mixing 0.1 nmol of purified human C3 (Teco Medical) with 2 nmol of DyLight 550 NHS Ester (Thermo Fisher) according to the manufacturer’s instructions and excess dye was removed with spin columns (cut-off 5 k). The efficiency of the labelling was then evaluated by SDS-PAGE (Supplementary Fig. 8).

### Statistical analyses

For cell culture studies, at least three independent experiments were carried out. Microscopic analyses were performed at least in duplicate and at least ten random fields were imaged per sample. Fluorimetry analyses were performed at least on six culture wells. Data are presented as mean ± SEM. Differences between groups were tested for statistical significance an unpaired two-tailed Student’s t test. P < 0.0001 was considered statistically highly significant and P < 0.005 was considered statistically significant.

## Electronic supplementary material

Supplementary Information

Supplementary Table S1

Supplementary Table S2

Supplementary Table S3

Supplementary Table S4
