# Supplementary material for: Complement C3 of the innate immune system secreted by muscle adipogenic cells promotes myogenic differentiation
Source: Sci Rep. 2017 Mar 13;7:171. doi: 10.1038/s41598-017-00099-7 (PMC5428314; doi:10.1038/s41598-017-00099-7)
Supplement: Supplementary file 1 — Supplementary Information [file 41598_2017_99_MOESM1_ESM.pdf]

Complement C3 of the innate immune system secreted by muscle adipogenic cells promotes myogenic differentiation

Thierry Rouaud†, Nader Siami†, Tanaelle Dupas†, Pascal Gervier, Marie-France Gardahaut, Gwenola Auda-Boucher* and Christophe Thiriet*

UFIP UMR-CNRS 6286, Epigénétique: prolifération et différenciation, Faculté des Sciences et des Techniques, 2 rue de la Houssinière, 44322 Nantes.

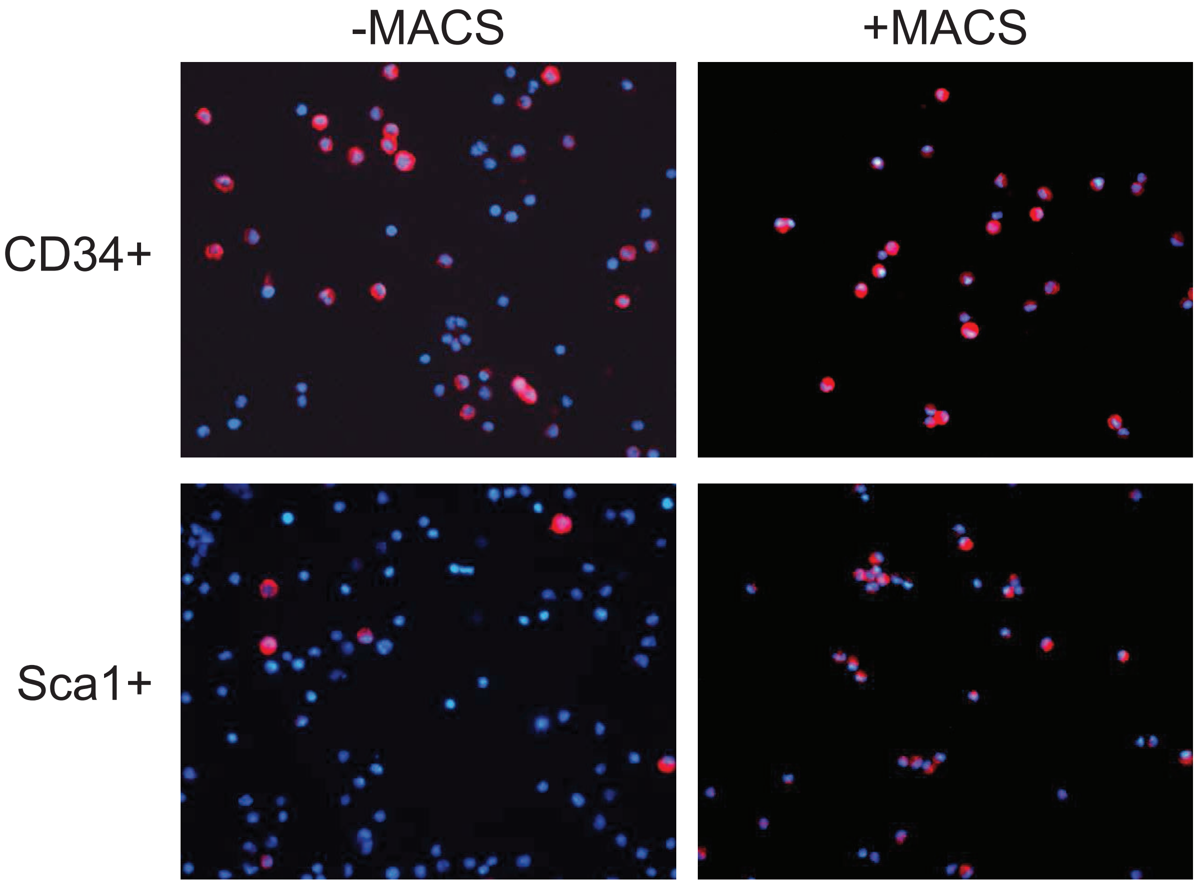

Supplementary Figure 1: Analyses of the efficiency of MACS. Initial fraction cells were isolated and immunostained with CD34 antibody (CD34+) and Sca1 antibody (Sca1+) prior to be subjected to magnetic sorting (-MACS) and after magnetic sorting (+MACS), respectively. Note that more than 90% of cells are immunostained after MACS.

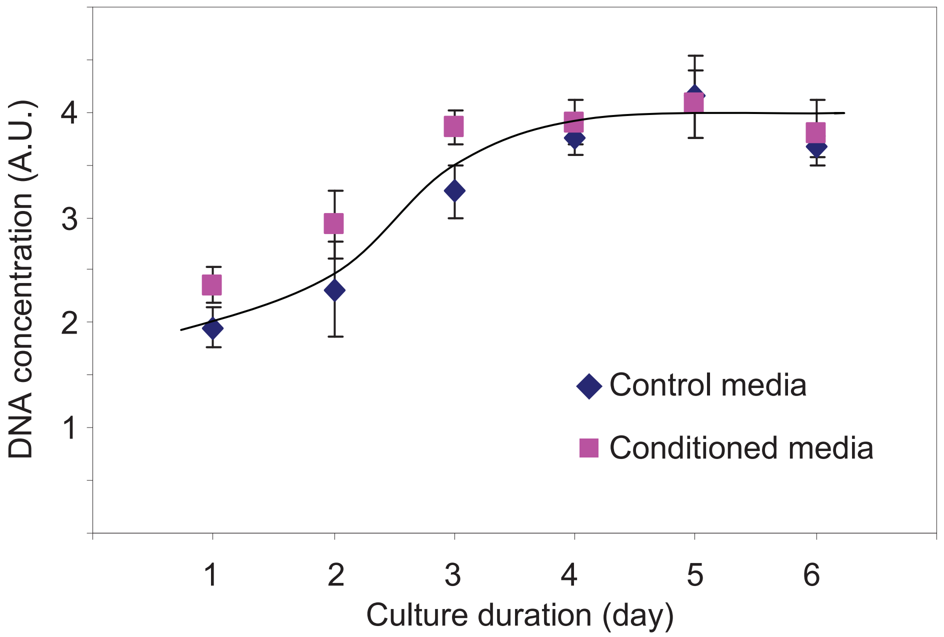

Supplementary Figure 2: Proliferation kinetics of initial fraction cells in control media and in conditioned media. The proliferation of the initial fraction cells in control media and in conditioned media was determined by measuring the DNA concentration in the cultures by fluorimetry.

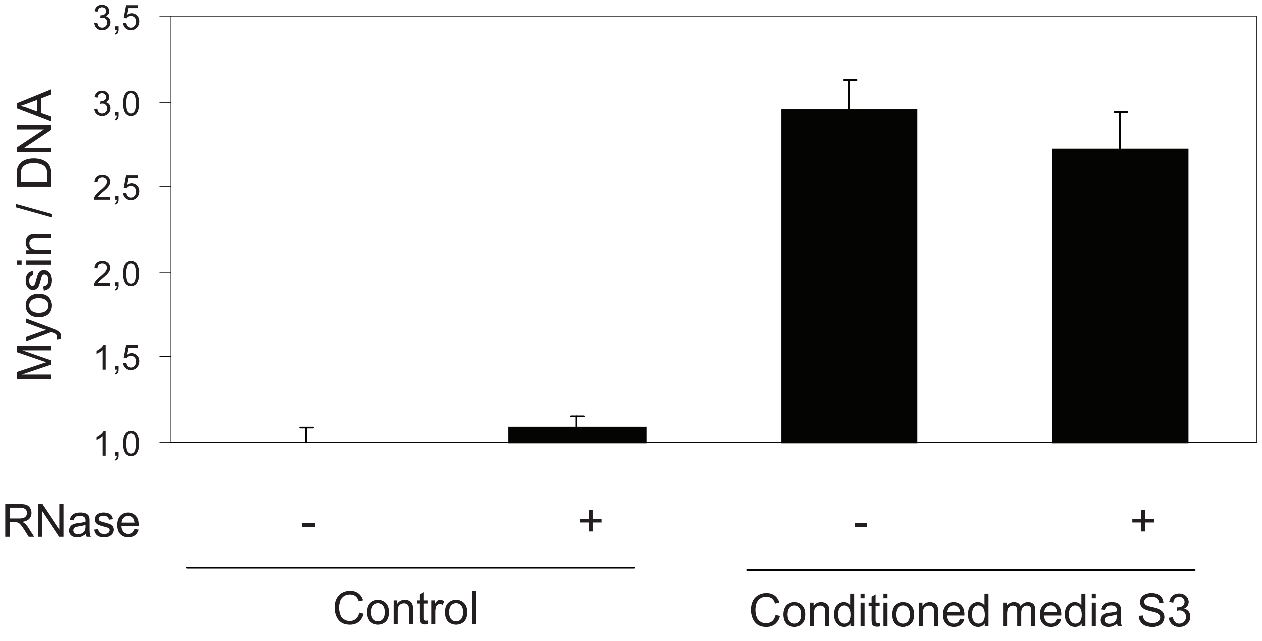

Supplementary Figure 3: Conditioned media are unaffected by RNase treatment. Conditioned media S3 and DMEM-FCS (Control) were untreated (-) and treated (+) with RNase A. Myogenic differentiation induced by the culture media was then assessed on primary cells.

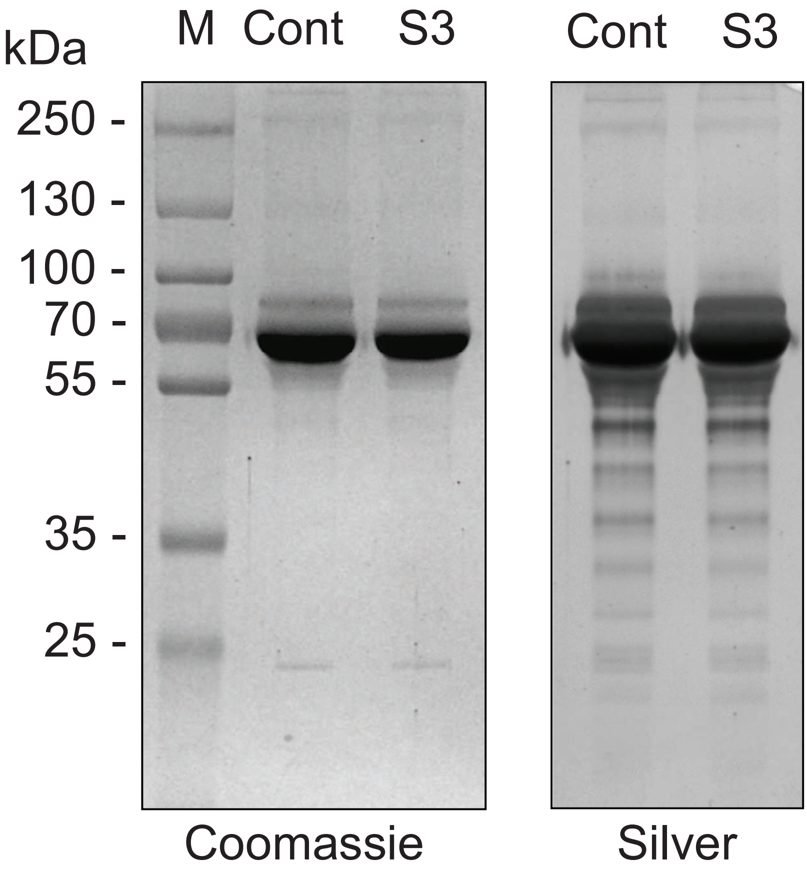

Supplementary Figure 4: Sca1 cells secrete proteins in low abundance. DMEM-FCS (Cont) and conditioned media S3 were resolved in SDS-PAGE. Gel staining with Coomassie Brilliant Blue (Coomassie) and by silver staining (Silver) failed to detect specific proteins.

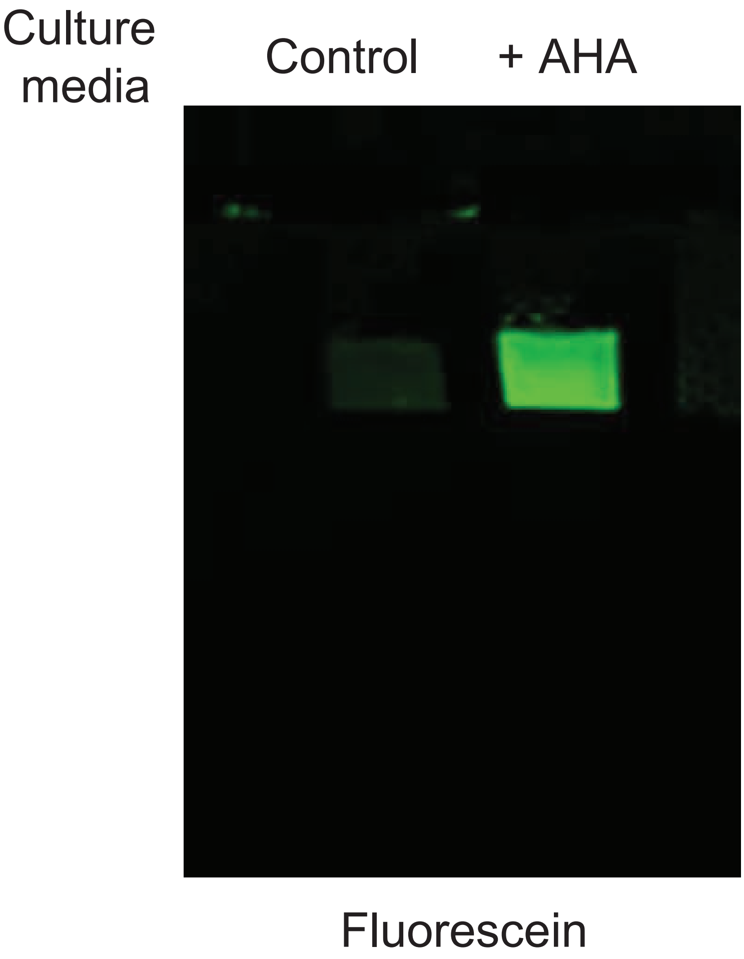

Supplementary Figure 5: Control of the specificity of the purification of soluble factors from Sca1 culture media. Sca1 primary cell culture media, in the absence of AHA (Control) and in the presence of AHA (AHA), respectively, were coupled to DBCO magnetic beads by click chemistry and exhaustively washed. Magnetic beads were then incubated with fluorescein-5-maleimide and loaded onto SDS-PAGE. Fluorescein-coupled proteins covalently attached to beads were visualised by UV illumination.

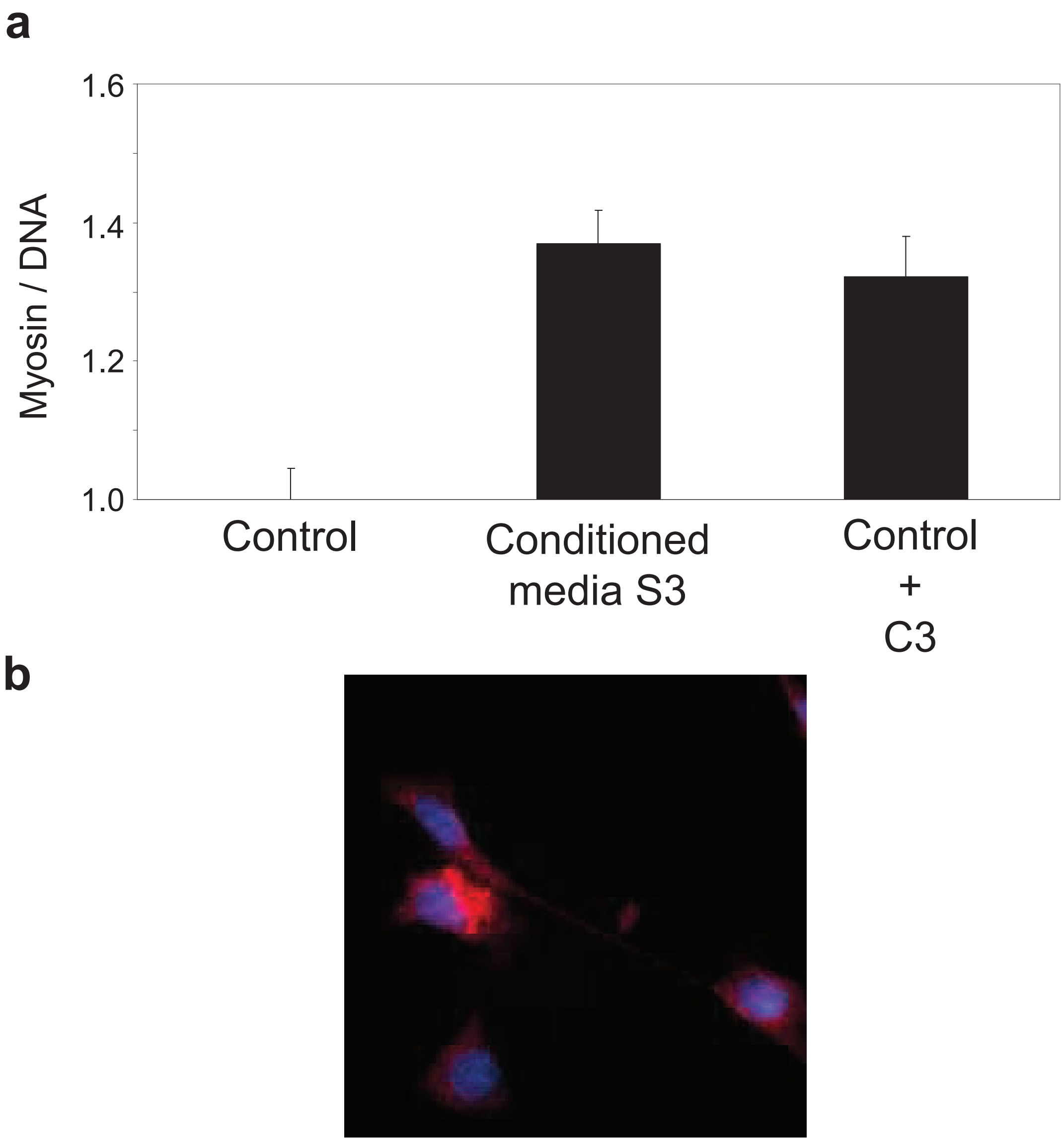

Supplementary Figure 6: Myogenic differentiation of C2C12 cells is enhanced by internalization of C3. (a) C2C12 cells were cultured in DMEM-10%FCS (Control), conditioned media S3 (conditioned media S3), and control media supplemented with C3 (control + C3). Myogenic differentiation of the cultures was determined after 5 days relative to the control indexed to 1. (b) Fluorescently labeled C3 was supplemented to the control media of C2C12 cell culture. After 4h, cells were observed by fluorescent microscopy.

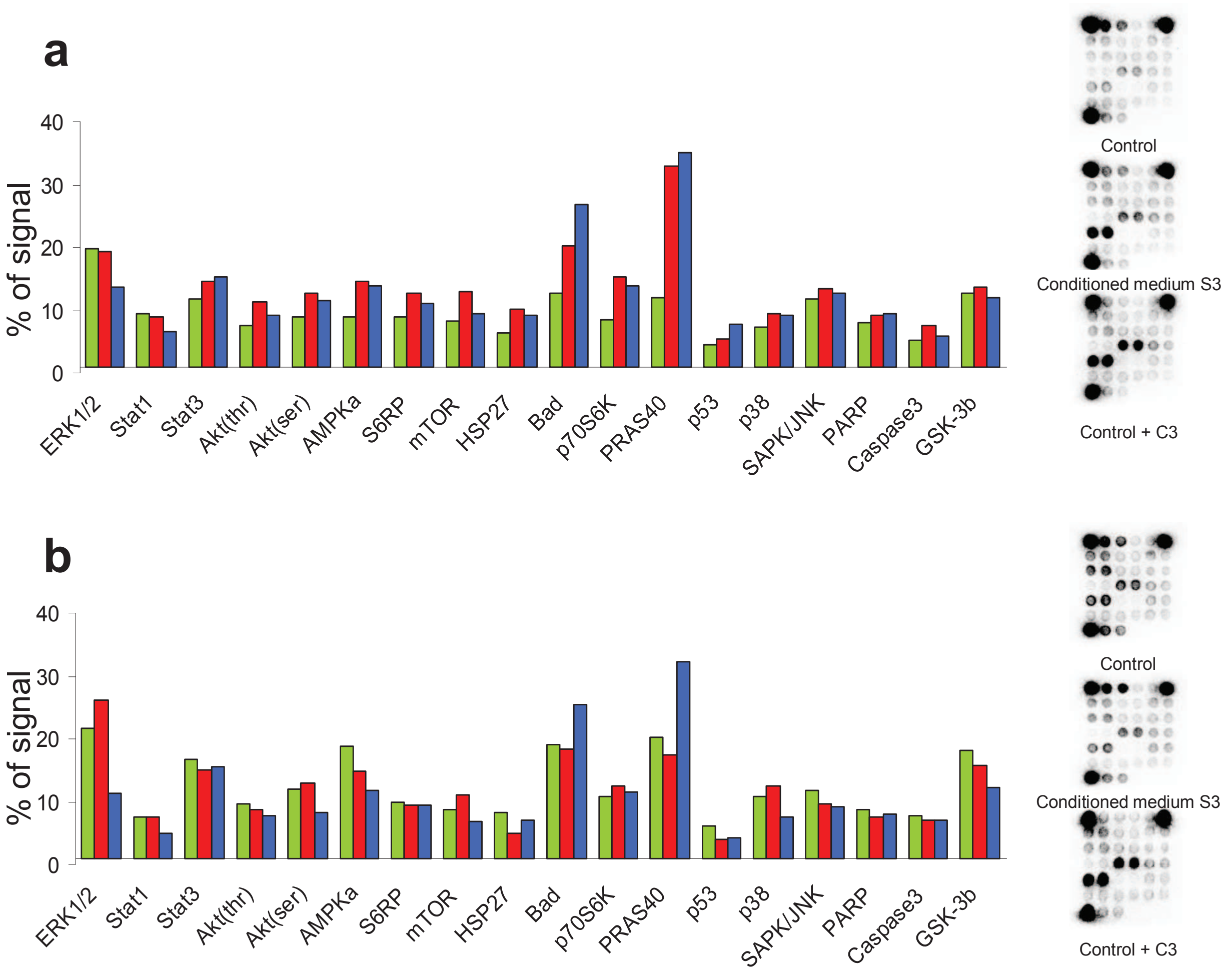

Supplementary Figure 7: Myogenic differentiation by conditioned medium and C3 do not reveal signalling pathway activation. Primary cells cultured in DMEM-FCS (Control, green), conditioned medium S3 (Conditioned medium S3, red) and DMEM-FCS supplemented with C3 (Control +C3, blue) were analysed using antibody arrays for intracellular signalling pathways (PathScan Intracellular Signaling Array Kit, Cell Signaling) after 2 days of culture (a) and 4 days of culture (b), respectively.

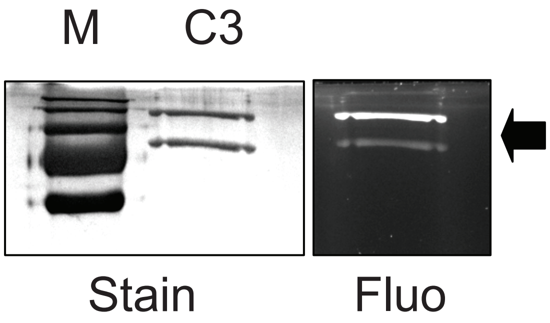

Supplementary Figure 8: Fluorescence labelling of C3. Purified human C3 was mixed with fluorescent DyLight 550 NHS Ester for 30 min. The efficiency of the labelling was evaluated by SDS-PAGE.
